# Species‐Specific Optimisation and Environmental Regulation of Biofilm Formation in *Enterobacter cloacae* : Inhibitory Role of Glucose in Biofilm Development

**DOI:** 10.1111/1758-2229.70347

**Published:** 2026-05-22

**Authors:** Srishti Singh, Virendra Bahadur Yadav, Alok Kumar Singh, Gopal Nath, Sasmita Chand, Nirmalendu Sekhar Mishra, Jagdeep Kumar Nayak

**Affiliations:** ^1^ Department of Botany C.M.P. Degree College, University of Allahabad Prayagraj India; ^2^ Department of Microbiology Institute of Medical Sciences, Banaras Hindu University Varanasi India; ^3^ Viral & Research Diagnostic Laboratory Institute of Medical Sciences, Banaras Hindu University Varanasi India; ^4^ Manipal School of Architecture and Planning, Manipal Academy of Higher Education Manipal India; ^5^ Green Energy & Applied Research (G.E.A.R) Lab, Department of Environmental Science and Engineering SRM University‐AP Amaravati India; ^6^ Department of Chemical Sciences University of Limerick Limerick Ireland

**Keywords:** 96‐well microtiter plate assay, biofilm classification, biofilm formation, drug‐resistant infections, *Enterobacter cloacae*

## Abstract

Bacterial biofilms are prevalent in clinical environments, contributing to persistent infections associated with medical devices. *Enterobacter cloacae* forms biofilms on nonliving surfaces, leading to drug‐resistant, recurrent infections that are difficult to treat. Biofilm development in *Enterobacter* species, including *E. cloacae* , occurs through five stages: reversible attachment, irreversible attachment, microcolony formation, maturation and dispersal. Initial attachment is mediated by adhesins, including fimbriae and lipopolysaccharides, which interact with surfaces. This is followed by secretion of an extracellular polymeric substance matrix composed of polysaccharides, extracellular DNA and proteins, providing stability and protection. This study aimed to establish a standardised in vitro 0.5% crystal violet staining method to quantify biofilm production in *E. cloacae* isolates and classify isolates by biofilm‐forming capacity. Biofilm was quantified by optical density at 570–600 nm. A 96‐well microtiter plate assay quantified biofilm formation in 40 *E. cloacae* strains collected between July 2021 and April 2023. Growth conditions were optimised, including culture media, fixation techniques and additive concentrations of glucose and sodium chloride. Brain heart infusion broth was optimal, and heat fixation was superior; glucose had no effect, whereas 1%–2% sodium chloride enhanced biofilm production. These findings improve understanding of environmental regulation of biofilm formation and microbial persistence across habitats.

## Graphical abstract

This study demonstrates that mild salinity stress (1%–2% NaCl) significantly enhances biofilm formation in *Enterobacter cloacae* . Using a standardised 96‐well microtiter plate assay, we optimised in vitro biofilm quantification and classification, providing a reproducible framework for studying environmental and clinical biofilm development mechanisms.

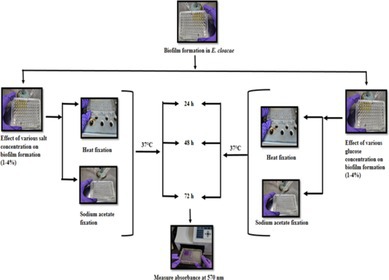

## 1 Introduction

*Enterobacter cloacae* , a Gram‐negative bacterium, is a member of the Enterobacteriaceae family (González‐Gómez et al. 2024). It inhabits diverse ecological niches and constitutes part of the normal gastrointestinal flora in 40%–80% of humans (Moreira De Gouveia et al. 2024; Paterson et al. 2005). However, the clinical significance of *E. cloacae* arises from its ability to colonise in hospital environments and medical devices, where it forms biofilms on catheters and prosthetics, leading to opportunistic infections such as bloodstream infections, pneumonia, urinary tract infections, wounds, skin and soft tissue infections and ocular complications (Anju et al. 2020; Storti et al. 2005). Bacterial biofilms are widely recognised as contributors to cystic fibrosis, periodontitis and hospital‐acquired infections associated with prosthetic heart valves and catheters (Datta et al. 2024; Delle Bovi et al. 2011). Biofilms comprise bacterial colonies surrounded by a matrix that adheres to surfaces and to other bacteria. Research indicates that biofilm bacteria are abundant in nearly all ecosystems, both in numbers and metabolic activity, making them challenging to eliminate (Charlton et al. 2022; Ragupathi et al. 2024). A biofilm comprises cells that attach to a surface and are enveloped by a mucous extracellular matrix of proteins, nucleic acids and polysaccharides. Most bacteria exist either as individual planktonic cells or as components of biofilms (Flemming et al. 2016; Flemming and Wuertz 2019). Biofilm formation is a defensive mechanism against harmful environmental conditions, including temperature fluctuations, pH changes, oxygen scarcity, dehydration, metal toxicity acid exposure, increased salinity and nutrient deficiency (Carezzano et al. 2023; Pal et al. 2022). The primary function of the biofilm matrix is to protect bacterial communities from harmful effects, whether from internal factors (e.g., the innate immune response) or from external factors (e.g., UV exposure and antibiotics). Biofilms are implicated in approximately 80% of human bacterial infections, increasing morbidity and mortality rates (Römling and Balsalobre 2012; Schulze et al. 2021). Bacteria that can form biofilms survive at rates 10–1000 times higher than their free‐floating counterparts and demonstrate significant treatment resistance. Three key elements collaborate to form biofilms: bacterial cells, the surface to which they attach and the surrounding environment (Sauer et al. 2022; Van Houdt and Michiels 2010). Nevertheless, environmental factors such as temperature, pH, osmolality, O_2_ levels, nutrient availability and the presence of other bacteria also play a vital role (Stepanović et al. 2003). The patterns and behaviours of biofilm production by a specific bacterium are influenced by various factors (Goller and Romeo 2008). Consequently, biofilms exhibit significant diversity and uniqueness, both among themselves and within the environments in which they develop. This variability complicates accurate in vitro characterisation of biofilms, making it essential to establish laboratory conditions that closely mimic the natural environments under investigation. The effectiveness of antibiotics is often limited by the presence of various species in this complex, poorly understood antibiotic‐resistant biofilm (Singh, Singh, Singh, Yadav, et al. 2025) (Jiang et al. 2020). Biofilm formation enhances bacterial survival in hospitals and on patients, thereby increasing the risk of nosocomial infections (Assefa and Amare 2022). Furthermore, numerous studies have demonstrated that horizontal gene transfer is linked to biofilm formation (Madsen et al. 2012; Michaelis and Grohmann 2023). Therefore, biofilms are highly diverse, not only in the microorganisms they contain but also in the specific environments in which they form. *E. cloacae* closely resembles other members of the family Enterobacteriaceae, including *Salmonella enterica* . Biofilm development in *S. enterica* serovar Typhi contributes to chronic typhoid carriage by enabling survival in the gallbladder despite antibiotic treatment (Nath et al. 2022). Such biofilms serve as reservoirs for infection, facilitating relapse and transmission. Recent bacteriophage therapy research has shown that phages can infiltrate *Salmonella* biofilms and lyse embedded cells, indicating a viable antibiofilm technique (Yadav and Nath 2022). A phage‐antibiotic combination also serves as an effective therapeutic option for eradicating biofilms in *E. cloacae* (Singh, Singh, Yadav, et al. 2025). Phage cocktails are also effective in urinary tract infections caused by drug‐resistant *E. cloacae* (Singh, Singh, Singh, Singh, et al. 2025). Although research on *E. cloacae* has been limited, our findings offer new approaches to managing its biofilm‐associated persistence. In light of these perspectives, this study assessed how varying concentrations of salt and sugar over time influence the attachment and biofilm formation of *E. cloacae* strains at body temperature (37°C) in brain–heart infusion (BHI) medium. BHI was selected as a nutrient‐rich laboratory medium commonly used in biofilm studies (Kowalska et al. 2020). We have used Sodium chloride and Glucose as the two standardised criteria because they directly probe the two key environmental cues that drive biofilm formation. Glucose serves as a primary carbon/energy source; its concentration regulates metabolic activity and, consequently, the rate at which cells produce extracellular polymeric substances (EPS) that hold the biofilm together (Misra et al. 2022). NaCl modulates osmotic pressure, thereby influencing cell turgor and stress‐response pathways that are known to upregulate biofilm‐related genes in many Gram‐negative bacteria (De Plano et al. 2025; Liu et al. 2023). By varying these two factors, we can systematically assess how nutrient richness and osmotic conditions together shape the biofilm's architecture and robustness, which is essential for reproducible standardisation across experiments.

Understanding biofilm regulation in *E. cloacae* has significance beyond clinical microbiology because this organism inhabits diverse environmental reservoirs including soil, wastewater, aquatic systems, plant‐associated niches and hospital effluents, where biofilm formation promotes persistence, nutrient acquisition and resistance to environmental stressors (Moreira De Gouveia et al. 2024; Flemming et al. 2016). Investigating how carbon availability and osmotic stress influence biofilm development therefore contributes to a broader understanding of microbial ecological adaptation, surface colonisation and survival strategies in environmentally fluctuating habitats.

## 2 Materials and Methods

The study was conducted in the Department of Microbiology at the Institute of Medical Sciences, Banaras Hindu University, Varanasi.

### 2.1 Isolation and Characterisation of Clinical Isolates

Forty isolates of *Enterobacter* species were obtained using standard microbiological techniques from various clinical specimens, including blood, pus, urine, sputum, body fluids and stool. These isolates were subjected to PCR‐based species discrimination using species‐specific primers. The identification of the isolates at the species level was accomplished using EC‐F (5′‐TGAAAACCTTATCCGCGA‐3′) and EC‐R (5′‐GGCAGGCTGGAAGATAAA‐3′) primers (Ji et al. 2021). The positive control used was *E. cloacae subsp. cloacae* ATCC 13047 type strains. For the reaction mixture, MQW (13.57 μL), 2.5 μL of 10X buffer, 2 μL of dNTPs, 0.8 μL of each primer (forward and reverse), 5 μL of DNA template and 0.33 μL of Taq Polymerase were combined for a single PCR reaction (25 μL). In summary, denaturation was performed at 94°C for 30 s, followed by annealing at 50°C for 30 s, an extension phase at 72°C for 1 min and a final extension at 72°C for 5 min. The amplified product was analysed using 1% agarose gel electrophoresis (GeNei TM Sl. No‐07/19/F/328, Peenya, Bangalore, India) (Singh, Singh, Singh, Yadav, 2025).

### 2.2 Bacteria Used for Biofilm Standardisation

In this study, a well‐characterised *E. cloacae* strain is used to assess biofilm formation and standardisation (Singh et al. 2017).

### 2.3 Cultivation Conditions for Biofilm Standardisation

All standard procedures utilised sterile BHI broth. To enhance standardisation, the following three conditions were applied:

#### 2.3.1 Supplementations With Sugar and Salt

Various concentrations of glucose and NaCl were utilised as supplements in BHI broth media (Table 1) (Singh, Singh, Yadav, et al. 2025).

**TABLE 1:** Different supplement concentrations are used in biofilm standardisation.

S. No.	Supplement	Concentration (%)
1	Glucose	0.5%
2		1%
3		2%
4		3%
5		4%
6	NaCl	0.5%
7		1%
8		2%
9		3%
10		4%

#### 2.3.2 Incubation Time

Incubation time was an important factor in biofilm formation and was set at 24, 48 and 72 h to evaluate its effects (Singh, Singh, Yadav, et al. 2025).

#### 2.3.3 Fixation Condition

We employed two methods to standardise fixation. The first involved thermal fixation, wherein samples were incubated at 65°C for 15 min (Singh et al. 2017). The second utilised a chemical fixation method using 2% (w/v) sodium acetate, incubated for 30 min at 37°C. Following chemical fixation, the plates were washed three times with 1× phosphate‐buffered saline (PBS) to remove residual fixative, then air‐dried at 37°C before staining.

#### 2.3.4 Procedure for Biofilm Formation

Biofilm formation was utilised in Thermo Scientific Nunc Micro Well 96‐well flat‐bottom microplates. A single colony of *E. cloacae* from a pure culture was inoculated in BHI broth and incubated at 37°C until the absorbance (λ600) reached 0.25. Each microplate well was filled with 190 μL of BHI broth and supplemented with the appropriate concentration of sugar or salt. A volume of 10 μL of the bacterial culture was added and incubated for a specified duration at 37°C in the incubator. After incubation, the media were discarded and the wells were washed three times with 1× PBS (pH 7.4) to remove planktonic bacteria, followed by predetermined fixation steps. The wells were washed three times with PBS and stained with crystal violet (0.5%) for 15 min. Next, the plate was rinsed under running water until all unbound dye was removed. 175 μL of absolute ethanol was applied, and the mixture was incubated at room temperature for 30 min to elute. The resulting eluates were transferred and resuspended in a fresh tissue culture plate. The optical density (OD) was measured using an ELISA plate reader at λmax 570 nm, with gentle shaking performed before reading.

All experiments were performed in triplicate, with each replicate paired. The data were expressed as mean ± standard deviation (SD). The optical density cut‐off (ODC) was determined from the mean OD of the negative control (BHI broth only) plus three SDs. Based on these values, the biofilm‐forming capacity was classified as follows: **Weak biofilm former:** ODC < OD ≤ 2 × ODC, **Moderate biofilm former:** 2 × ODC < OD ≤ 4 × ODC, **Strong biofilm former:** OD > 4 × ODC (Metwally et al. 2019; Nyenje et al. 2013).

## 3 Biofilm Evaluation by Standard CFU (Colony Forming Unit) Count Against *E. cloacae*

The viable cell count in a biofilm indicates the number of living cells capable of growing and reproducing within it. To determine this, the biofilm layers are typically removed from the surfaces by scraping, mechanically disrupted to achieve uniform dispersion, diluted in Normal Saline and then cultured on agar. The colonies that form after incubation reflect the number of viable cells originally present within the biofilm. This CFU enumeration method remains a standard quantitative approach for assessing viable cell populations associated with biofilms (Welch et al. 2012).

## 4 Results

### 4.1 Identification of Bacterial Isolates

**FIGURE 1:**
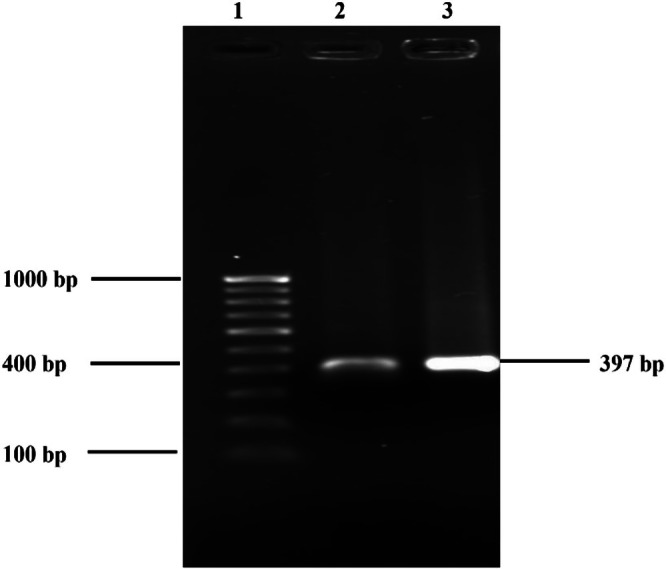
Gel image showing molecular identification using a species‐specific primer Lane 1: 100 bp Ladder, Lane 2: ATCC 13047 *Enterobacter cloacae* *subspecies cloacae* (Positive control), Lane 3: Clinical isolates ( *E. cloacae* ) (Singh, Singh, Singh, Singh, et al. 2025).

A total of 40 *Enterobacter* isolates were identified and classified as *E. cloacae* using species‐specific primers targeting aminocyclase, EC‐F (5′‐TGAAAACCTTATCCGCGA‐3′) and EC‐R (5′‐GGCAGGCTGGAAGATAAA‐3′), as shown in Figure 1.

### 4.2 Biofilm Formation Among 40 *Enterobacter* Isolates

The average optical density (ODavg) of the negative controls was 0.108 ± 0.0098. As a result, an OD cutoff value of 0.137 was set. After calculating the OD average and cutoff values, the ODS of *E. cloacae* were categorised according to the standards in Tables 2 and 3.

**TABLE 2:** Criteria for Biofilm classification and source of isolation (Singh et al. 2017).

Classification criteria	Optical density measurements	Total isolates	% of isolates
OD ≤ OD_cut_ = Nonbiofilm former (NB)	OD is < 0.137	6/40	15%
OD_cut_ < OD ≤ 2 × OD_cut_ = Weak biofilm former (WB)	0.137 < OD ≤ 0.274	8/40	20%
2 × OD_cut_ < OD ≤ 4 × OD _cut_ = Moderate biofilm former (MB)	0.274 < OD ≤ 0.548	20/40	50%
OD > 4 × OD_cut_ = Strong biofilm former (SB)	OD > 0.548	6/40	15%

**TABLE 3:** Species‐wise distribution of *Enterobacter cloacae* among different clinical samples (Singh, Singh, Singh, Yadav, et al. 2025).

Clinical samples	*E. cloacae complex n* = 40
Urine	18 (45%)
Pus	15 (37.5%)
Sputum	3 (7.5%)
Body fluids	3 (7.5%)
Blood	1 (2.5%)

### 4.3 Effect of Incubation Time

Biofilm formation occurred over an incubation period of 24–72 h. A 24‐h incubation had no significant effect on biofilm development. The highest biofilm biomass was observed after 72 h of incubation, particularly under mild salt stress.

### 4.4 Fixation Condition

Heat fixation and sodium acetate fixation were employed. After 72 h of incubation under both conditions, the highest biofilm formation occurred under mild salt stress. When comparing the two fixation methods, heat fixation yielded the most effective visualisation of biofilm formation after 72 h in NaCl‐enriched broth.

### 4.5 Effect of Sugars

**FIGURE 2:**
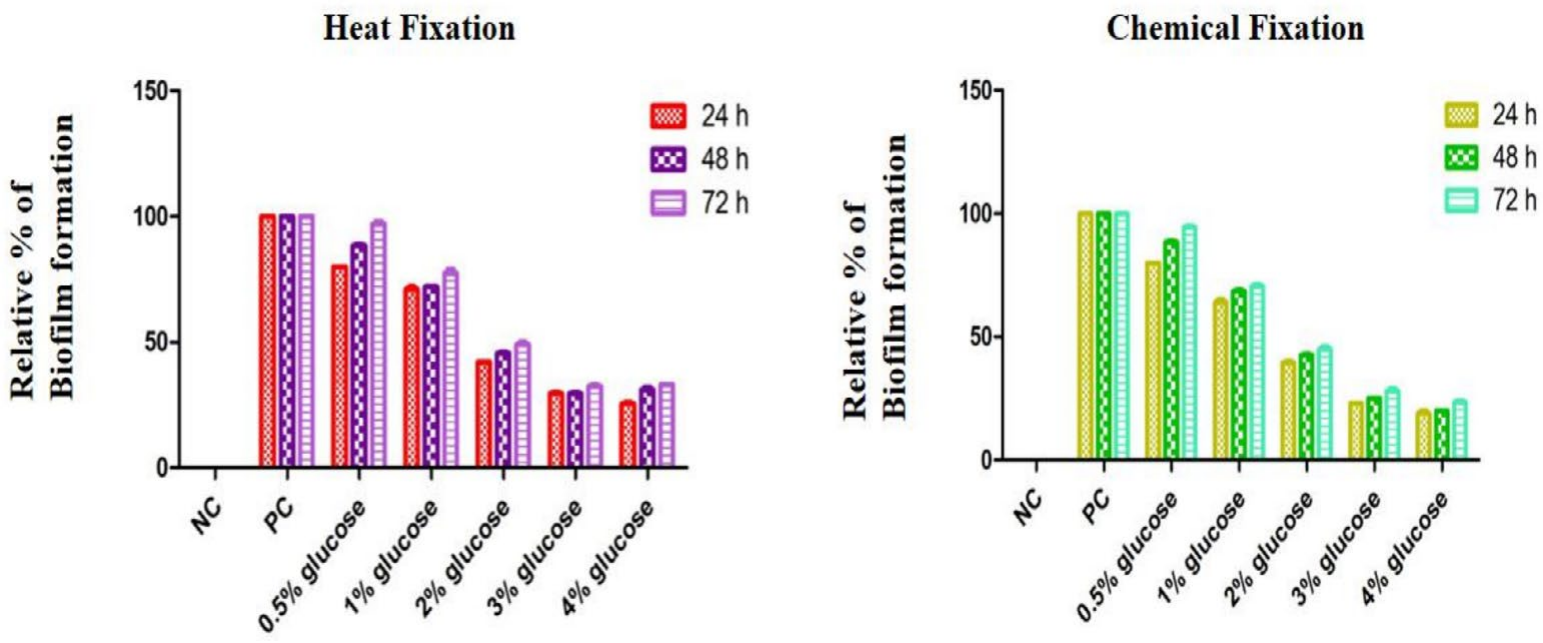
Effect of different sugar concentrations on biofilm formation in *Enterobacter cloacae* under both conditions (Heat Fixation vs. Chemical Fixation) at various incubation times (*p* < 0.0001). (NC = Negative Control; PC = Positive Control).

Adding sugars (glucose) to the medium significantly influenced *E. cloacae's* ability to form biofilms. When glucose was supplemented, the absorbance decreased markedly compared to the positive control (broth without supplementation) (Figure 2).

### 4.6 Effect of Salt Stress

**FIGURE 3:**
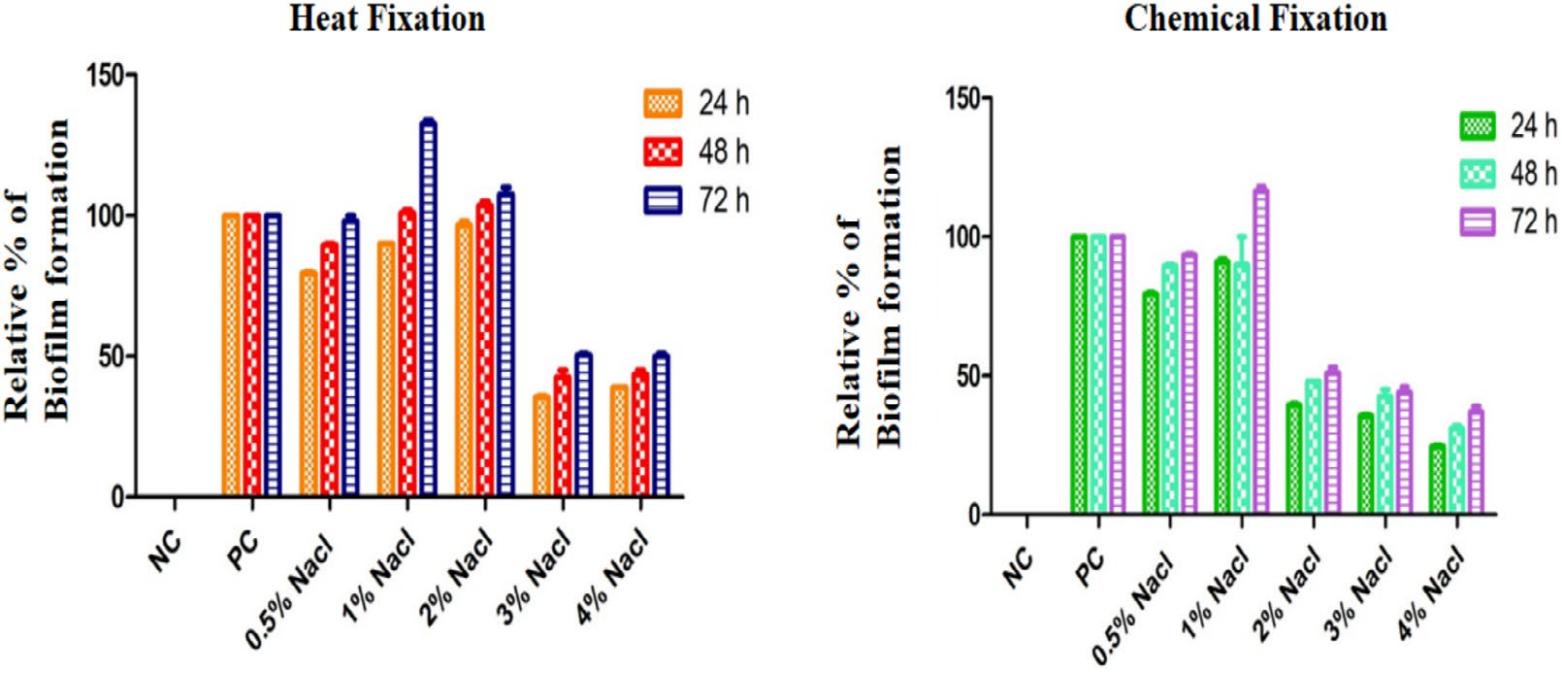
Effect of different salt concentrations on biofilm formation in *Enterobacter cloacae* in both conditions (Heat fixation vs. Chemical Fixation) at various incubation times (*p* < 0.0001). (NC = Negative Control; PC = Positive Control).

Salt supplementation significantly enhanced biofilm formation as the concentration increased. Biofilm formation significantly increases at 1%–2% NaCl concentration. However, this ability declined rapidly once it reached a threshold level, regardless of other variables (Figure 3).

### 4.7 Evaluation of Viable Cell Count via Standard CFU Count in Different Treatment Orders

#### 4.7.1 Viable Count at Different Concentrations of Sodium Chloride in *E. cloacae*

**FIGURE 4:**
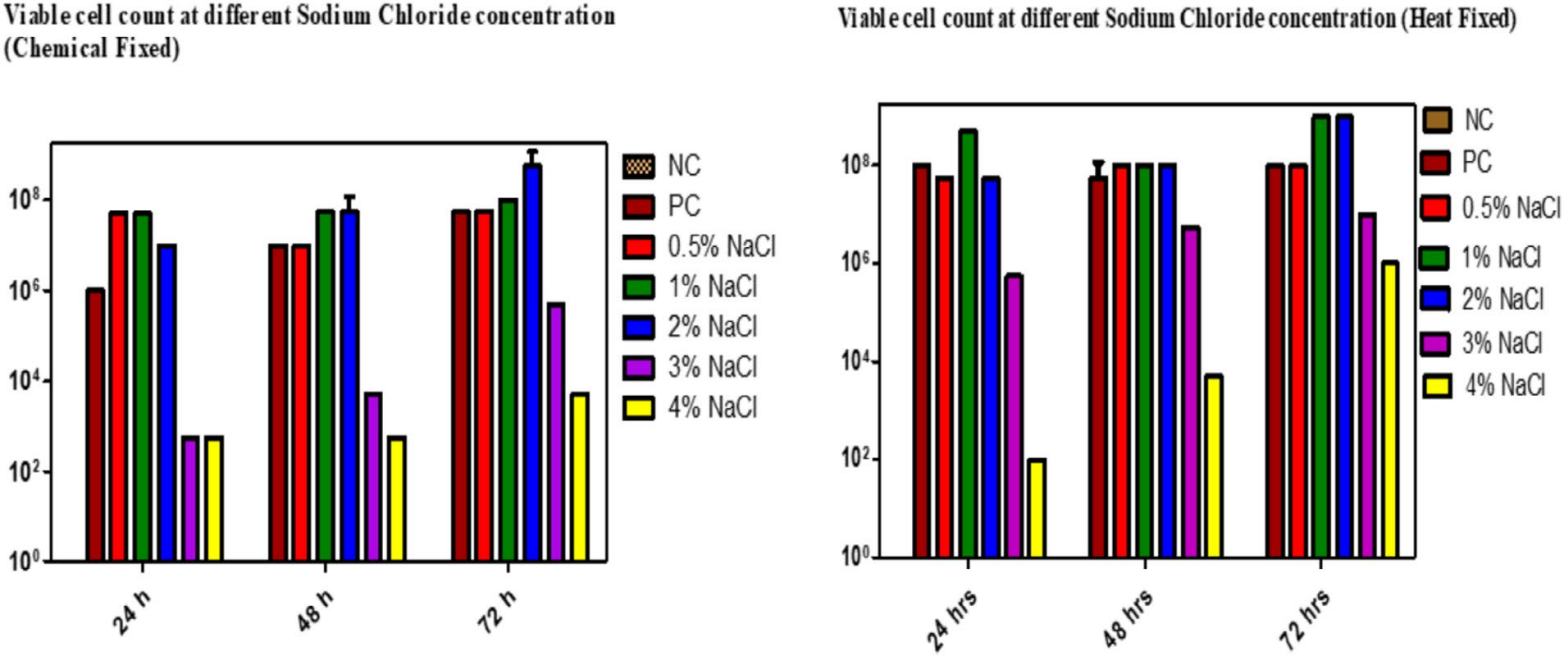
Viable cells count at different salt concentrations on biofilm formation in *Enterobacter cloacae* in both conditions (Heat fixation *p* value‐ 0.19 versus Chemical Fixation *p* value‐ 0.34) at various incubation times.

In this in vitro study, sodium chloride concentrations were used to standardise biofilm formation in *E. cloacae* , ranging from 0.5% to 4%. The addition of sodium chloride significantly affected the biofilm‐forming ability in *E. cloacae*. When comparing the fixation conditions and incubation time at different NaCl concentrations, the most effective biofilm was observed after 72 h of incubation under mild salt stress. The viable count increases by 1 log at 1%–2% NaCl concentration in heat fixation conditions, whereas no increase in viable count is observed during chemical fixation steps. Furthermore, at a 3%–4% NaCl concentration, a decrease in bacterial counts was observed within 72 h. ultimately, no increase in viable count was seen during a 24 to 48‐h incubation period (Figure 4).

#### 4.7.2 Viable Count at Different Concentrations of Glucose in *E. cloacae*

**FIGURE 5:**
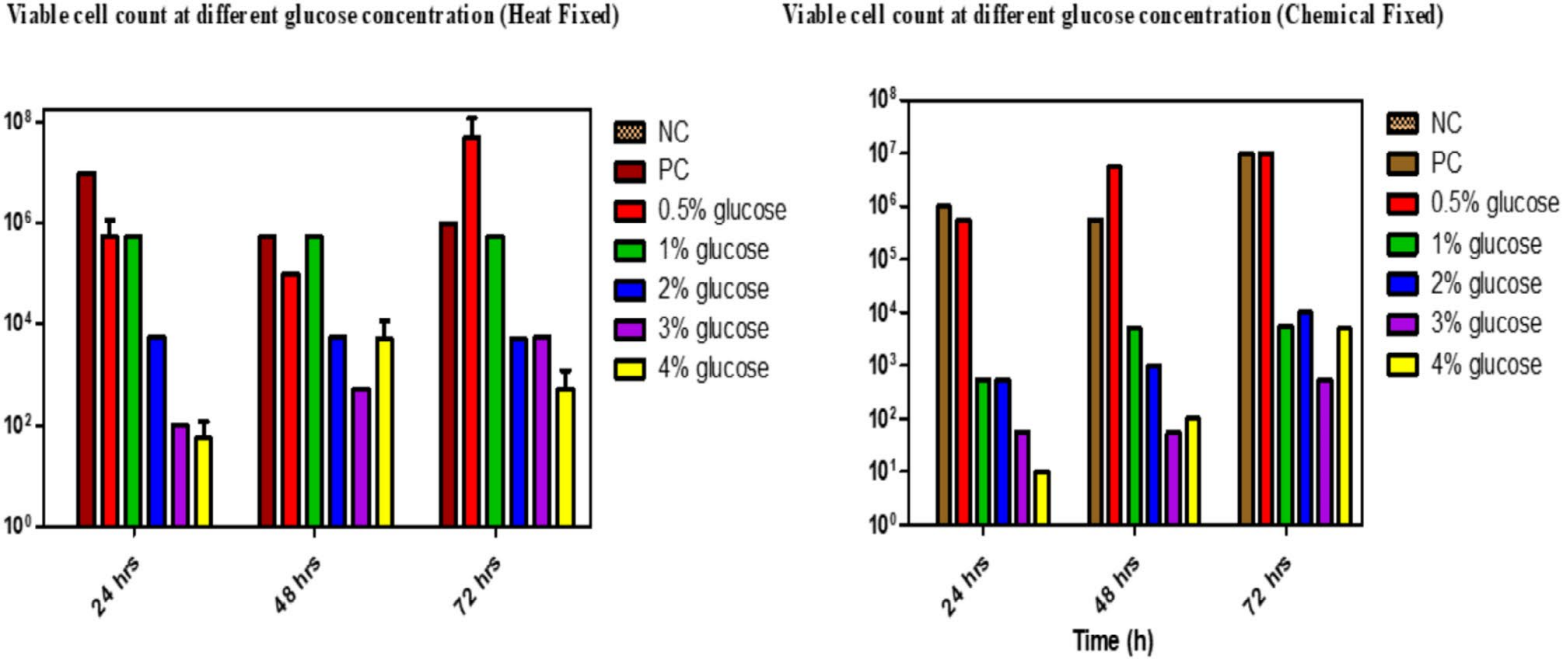
Viable cells count at different Glucose concentrations on biofilm formation in *Enterobacter cloacae* in both conditions (Heat fixation *p* value‐0.31 vs. Chemical Fixation *p* value‐0.33) at various incubation times.

In this in vitro study, different concentrations of glucose were used to standardise the biofilm of *E. cloacae* , ranging from 0.5% to 4%. The addition of glucose inhibited the biofilm‐forming ability of *E. cloacae* . The viable cell count increased by 1 log at 0.5% glucose concentration under heat fixation conditions, while a 4‐log reduction in viable cell count was observed at 1%–3% glucose concentration (Figure 5).

## 5 Discussion

Biofilms are structured microbial communities in which cells adhere to biotic or abiotic surfaces and become embedded within a self‐produced extracellular polymeric matrix. This matrix consists primarily of EPS, exopolysaccharides, fibrillar proteins and extracellular DNA, which collectively provide structural integrity and protection to microbial populations (Bremer and Krämer 2019). A significant proportion of EPS exists in hydrated forms, although hydrophobic structural components such as cellulose are also produced by several microorganisms (Dewasthale et al. 2018; Flemming et al. 2025). Biofilm formation is a dynamic and sequential process involving reversible attachment, irreversible adhesion, microcolony formation, maturation and dispersal, allowing microorganisms to adapt effectively to environmental fluctuations (Misra et al. 2022). During early colonisation, microbial cells interact with surfaces through hydrophobic, electrostatic and van der Waals forces, facilitating the transition from planktonic growth to a sessile lifestyle (Fazeli‐Nasab et al. 2022).

The ability of *E. cloacae* to form biofilms on plastic and other abiotic surfaces presents considerable concerns in both healthcare and industrial environments, particularly due to its persistence on medical devices and food‐processing surfaces. Previous studies demonstrated that all tested *E. cloacae* strains adhered successfully to microtiter plate wells after 48 h of incubation regardless of growth medium or incubation temperature, indicating a robust intrinsic biofilm‐forming capability (Nyenje et al. 2013). Additionally, BHI broth has previously been reported to support stronger biofilm formation than TSB in certain strains, which justified its selection as the growth medium in the present study for biofilm standardisation (Nyenje et al. 2013). Our findings further confirm that *E. cloacae* forms reproducible biofilms under laboratory conditions and that BHI provides a suitable nutrient‐rich environment for this purpose. Environmental conditions significantly influence biofilm development, and among these, temperature and incubation duration are major determinants. Prior studies have shown that prolonged incubation enhances biofilm maturation, with *E. cloacae* producing stronger biofilms after 24–48 h at 37°C (Nyenje et al. 2013). Elevated temperatures above 40°C have also been associated with enhanced biofilm formation in *Enterobacter* species (Iversen et al. 2004). Consistent with these observations, our results demonstrated maximal biofilm biomass after 72 h of incubation at 37°C, suggesting that prolonged incubation facilitates maturation and stabilisation of the biofilm architecture. This further supports the view that optimal biofilm development is highly dependent on both incubation time and strain‐specific physiological traits. Similar biofilm‐forming behaviour has been reported in related *Enterobacter* species including *E. hormaechei* (Liu et al. 2022). Importantly, the present findings contribute to our broader understanding of microbial environmental adaptation by demonstrating that biofilm formation in *E. cloacae* is highly responsive to physicochemical stressors. Biofilm development serves as an ecological survival mechanism that allows microorganisms to withstand nutrient limitation, osmotic stress, dehydration and other environmental pressures (Fazeli‐Nasab et al. 2022). The enhanced biofilm production observed under mild salt stress in this study suggests that *E. cloacae* employs biofilm formation as a protective adaptation to osmotic challenge, a strategy widely recognised among environmentally persistent microorganisms. Regarding fixation methodology, our study demonstrated that thermal fixation at 65°C for 15 min was superior to sodium acetate fixation for biofilm visualisation and quantification. Heat fixation effectively reduces bacterial viability, thereby simplifying downstream handling while minimising contamination risks (Mercer et al. 2017; Scher et al. 2005). Furthermore, thermal treatment promotes stabilisation of EPS and enhances adherence of the biofilm matrix to the substrate, reducing biomass loss during washing and staining procedures (Scher et al. 2005; Stobie et al. 2008). Consequently, heat fixation preserves the structural integrity of biofilms more effectively and improves crystal violet retention, producing clearer and more reproducible staining outcomes.

Although elevated temperature is used here primarily as a fixation step, heat exposure is also known to induce physiological stress responses in microorganisms. High‐temperature stress activates heat shock proteins, modifies membrane lipid composition and redirects metabolic pathways toward synthesis of protective molecules including EPS (Nandal et al. 2005; Niu and Xiang 2018). Such responses may partly explain the enhanced matrix stabilisation observed following thermal fixation. In this study, glucose supplementation markedly inhibited biofilm formation by *E. cloacae* , indicating that increased carbon availability suppresses sessile community development. This finding aligns with reports suggesting that the effects of glucose on biofilm formation vary among bacterial species and may either enhance or inhibit biofilm development depending on metabolic and regulatory pathways (Rode et al. 2007; Houot et al. 2010; Jahid et al. 2015). The observed reduction in biofilm formation may reflect the preferential use of glucose as a readily available carbon source, redirecting bacterial metabolism toward planktonic growth rather than EPS production and surface attachment. Moreover, glucose has been shown to suppress biofilm‐associated gene expression and EPS synthesis via catabolite repression pathways in certain bacteria (Rojo and Alejandro Dinamarca 2004). However, the precise molecular mechanism underlying glucose‐mediated inhibition in *E. cloacae* requires further validation.

From an environmental perspective, this glucose‐dependent repression suggests that nutrient‐rich environments may reduce the selective pressure for biofilm formation, whereas nutrient limitation may promote sessile adaptation as a survival strategy. Such metabolic flexibility likely contributes to the ecological success of *E. cloacae* across nutrient‐variable habitats including wastewater, host‐associated environments and contaminated surfaces. In contrast, sodium chloride supplementation enhanced biofilm formation at concentrations of 1%–2%, although this stimulatory effect declined at higher salt concentrations. Similar osmotic stress‐induced biofilm enhancement has been reported in several microorganisms including *Staphylococcus epidermidis* , *Clostridium ljungdahlii* and *Candida albicans* (Ferreira et al. 2021; Lee et al. 2014; Pemmaraju et al. 2016). Mild osmotic stress may stimulate EPS synthesis, enhance cell‐surface hydrophobicity and upregulate genes associated with adhesion and biofilm matrix production, collectively promoting surface colonisation (Lade et al. 2019). However, excessive osmotic stress may exceed the physiological tolerance of bacterial cells, leading to reduced viability and impaired biofilm formation. Our viable count data support this interpretation, as bacterial counts declined significantly at NaCl concentrations above 2%. High salt concentrations may also simulate physiologically relevant osmotic environments such as those encountered in the urinary tract, where *E. cloacae* frequently causes infection. Therefore, understanding salt‐mediated biofilm responses may have implications for studying bacterial persistence under clinically and environmentally relevant osmotic conditions (Ferreira et al. 2019; Kumar and Prasad 2025). Finally, quorum sensing (QS) is recognised as a major regulator of biofilm development and microbial collective behaviour, coordinating virulence, motility, antibiotic resistance and surface colonisation in response to environmental signals (Singh et al. 2024). Since QS pathways respond to external stressors including nutrient availability, salinity and temperature, future studies should investigate the relationship between osmotic regulation and QS‐mediated biofilm signalling in *E. cloacae* . Targeting QS pathways may represent an effective antibiofilm strategy, as disruption of signalling reduces pathogenicity without directly inhibiting bacterial growth (Rather et al. 2021). Overall, this study demonstrates that biofilm formation in *E. cloacae* is highly dependent on environmental and nutritional cues, highlighting the organism's adaptive capacity to modulate its lifestyle in response to physicochemical changes. These findings improve our understanding of how environmental stressors regulate microbial persistence and may inform future strategies aimed at controlling *E. cloacae* biofilms in both clinical and environmental settings.

## 6 Conclusions and Future Perspectives

The opportunistic pathogen *E. cloacae* can cause severe disease, typically nosocomial infections, including septicemia, pneumonia, urinary tract infections and soft‐tissue infections. *Enterobacter* infections are often regarded as a paradigm of hospital‐acquired infections. The indiscriminate use of antibiotics has led to a significant rise in outbreaks caused by microorganisms resistant to antimicrobial drugs, such as β‐lactamase‐producing *E. cloacae* . The study also indicated the suitability of BHI and TSB media for cultivating *E. cloacae* biofilms; however, temperature and incubation time significantly affected biofilm formation by these bacteria. Nosocomial *Enterobacter* infections significantly burden the economy and patients' life expectancy in developed countries. Therefore, advancing the prevention of hospital‐acquired infections will necessitate new strategies for infection control. The growing evidence of *E. cloacae's* ability to form biofilms, particularly on medical devices, along with recent data supporting a correlation between this behaviour and the acquisition of antibiotic resistance, should heighten awareness of the risks posed by this pathogen in hospital environments.

Exploring these virulence factors and studying new mechanisms to control them could be an important means of counteracting *E. cloacae* nosocomial infections. In particular, the biofilm mode of growth renders bacteria up to 1000 times more resistant to antibiotic therapy. In *E. cloacae* , numerous studies have been conducted to elucidate better the mechanisms underlying this resistance, demonstrating that the limitation of antibiotic molecule penetration through the biofilm matrix is not the primary cause of the increasing resistance; rather, it is the slow growth rate in the centre of the biofilm that contributes to this phenomenon. The formation and maintenance of biofilms have been extensively investigated in relation to environmental signals, metal ions and other physiological parameters. Additionally, the role of EPS in nutrient cycling, gene transfer, protection against antibiotics and immune function is significant. In any case, other mechanisms are involved, and further studies are warranted to address the challenge of developing new concepts for preventive measures against nosocomial *Enterobacter* infections. Identifying strategies and techniques that biofilms use to evade potent antibiotics and applying environmentally friendly biological, physical and chemical processes to disrupt biofilm communities are equally noteworthy. Using various physical, chemical or biological techniques, researchers are investigating the development of potent antibiofilm agents derived from natural products and/or combinations of phytochemicals. This strategy aims to demonstrate a synergistic effect without increasing microbial resistance. Further knowledge of mechanisms, signalling cascades, gene regulation and the roles of signalling molecules, including secondary messengers, in the formation, growth, maturation and dispersal of biofilms is urgently needed, as is the development of standardised antibiofilm protocols and the requirement for in vivo validation. Importantly, the present work also enhances understanding of how environmental stimuli regulate microbial community behaviour in opportunistic environmental bacteria. By demonstrating that osmotic stress promotes while glucose represses biofilm formation, this study highlights the role of nutrient and salinity gradients as ecological determinants of microbial surface colonisation. These findings may assist future studies investigating *E. cloacae* persistence in environmental reservoirs, wastewater systems, biofouling communities and host‐associated microbiomes.

## Author Contributions

**Virendra Bahadur Yadav:** writing – review and editing. **Nirmalendu Sekhar Mishra:** writing – review and editing. **Srishti Singh:** conceptualization, investigation, writing – original draft. **Sasmita Chand:** writing – review and editing. **Jagdeep Kumar Nayak:** writing – review and editing. **Gopal Nath:** supervision. **Alok Kumar Singh:** supervision.

## Funding

The authors have nothing to report.

## Ethics Statement

The authors have nothing to report.

## Consent

The authors have nothing to report.

## Conflicts of Interest

The authors declare no conflicts of interest.

## Data Availability

The data that support the findings of this study are available on request from the corresponding author. The data are not publicly available due to privacy or ethical restrictions.
